# The effects of differences in trophectoderm biopsy techniques and the number of cells collected for biopsy on next‐generation sequencing results

**DOI:** 10.1002/rmb2.12463

**Published:** 2022-04-20

**Authors:** Yamato Mizobe, Yukari Kuwatsuru, Yuko Kuroki, Yumiko Fukumoto, Mari Tokudome, Harue Moewaki, Mia Watanabe, Tokiko Iwakawa, Kazuhiro Takeuchi

**Affiliations:** ^1^ Takeuchi Ladies Clinic/Center for Reproductive Medicine Aira Japan

**Keywords:** euploidy, mosaic, next‐generation sequencing, preimplantation genetic testing, trophectoderm biopsy

## Abstract

**Purpose:**

To examine how differences in trophectoderm biopsy techniques affect the frequency of mosaic embryos and sequencing results.

**Methods:**

We examined differences in next‐generation sequencing (NGS) analysis results among operators or according to biopsy technique. Additionally, we determined the cut‐off for the number of collected cells to predict the occurrence of mosaicism. We collected cells according to the cut‐off value and examined whether there was a difference in the NGS analysis results between the pulling and flicking methods.

**Results:**

There was no difference in the NGS analysis results among the operators. Regarding re‐biopsy, changes in the mosaic were observed in all specimens. The cut‐off value for the number of collected cells was five, and when more than five cells were collected, there was no difference in the NGS analysis results between the two methods.

**Conclusions:**

We demonstrated that if trophectoderm biopsy techniques and NGS are stable, the cell collection location has a greater effect on NGS results than the biopsy technique.

## 1 INTRODUCTION

Assisted reproductive technology (ART), such as in vitro fertilization (IVF) and embryo transfer or intracytoplasmic sperm injection, has evolved since the birth of a baby through IVF in 1978.^1^ The rapid development of ART has led to the stabilization of pregnancy outcomes and diffusion of technology. Concurrently, with the development of genetic analysis technology, preimplantation genetic testing, wherein genetic diagnosis is performed from preimplantation embryos based on IVF technology, has been established. The mean age of female patients, who underwent ART in Japan, was 38 years.^2^ This mean age increases annually and is predicted to continue to increase.^2^ Aneuploidy, the presence of an abnormal number of chromosomes, becomes dramatically more common with increasing maternal age.^3^, ^4^ Testing for an abnormal number of chromosomes in an embryo is called preimplantation genetic testing for aneuploidy (PGT‐A), which has been demonstrated in many studies.^5^, ^6^, ^7^, ^8^, ^9^ Avoiding the transfer of aneuploid embryos can also reduce unnecessary abortions and is reported to shorten treatment cycles and increase cost‐effectiveness.^10^, ^11^, ^12^ Thus, PGT‐A is considered to have sufficient merit and has rapidly become widespread in ART, wherein patients continue to age.

Trophectoderm (TE) biopsy, through which multiple cell specimens can be harvested in the blastocyst stage, and comprehensive genetic analysis with next‐generation sequencing (NGS) have recently become the predominant method in PGT‐A. In PGT‐A, opinions vary greatly regarding the handling of mosaic embryos. A study reported that babies with normal karyotypes were born owing to the transfer of mosaic blastocysts,^13^ while another study that comprehensively examined 1000 mosaic embryos reported that many babies were born.^14^ However, another study claimed that blastocysts with ≥40% abnormal cells should not be prioritized for embryo transfer owing to low ongoing implantation rates.^15^ There is ongoing discussion that factors, such as patient characteristics and percentages of mosaicism, should be considered when determining whether to transfer mosaic embryos.^14^, ^16^ Mosaic embryos result from post‐fertilization errors in somatic cell division and, therefore, may not be affected by age^14^; rather, mosaic embryos may arise from TE biopsy techniques. There are reports that analysis results vary by center^17^, ^18^ and that the concordance rate of mosaic occurrence by re‐biopsy is low.^19^ Therefore, the present study examined how differences in TE biopsy techniques affect the frequency of mosaic embryos and analysis results. We also investigated how the number of cells collected after biopsy affected the results of NGS analysis. While collecting too few cells increases the likelihood of inaccurate analysis results, collecting too many cells further damages the embryo. Blastocysts that are not particularly high in quality are especially susceptible to damage. Therefore, we determined the optimal number of cells to be collected.

## 2 MATERIALS AND METHODS

### 2.1 Patients background

Patients with repeated ART failures were included in the study. Patients with chromosomal abnormalities were excluded. For example, patients who were eligible for preimplantation genetic testing for chromosomal structural rearrangements or preimplantation genetic testing for monogenic disorders were excluded from this study. The mean maternal and paternal age were 36.38 ± 2.90 years (range: 30–42) and 36.00 ± 4.27 years (range: 26–42), respectively.

### 2.2 Ovarian stimulation

Oocyte retrieval was performed at the Takeuchi Ladies Clinic between 2016 and 2020. In the oocyte retrieval cycle, the patients’ conditions were stimulated using a standard gonadotropin‐releasing hormone agonist, follicle‐stimulating hormone (FSH) protocols, or the antagonist FSH protocol. Oocytes were retrieved under transvaginal ultrasound guidance, 36‐h post‐injection of human chorionic gonadotropin (Fuji Pharmaceutical Company, Ltd.).

### 2.3 Embryo culture

Cumulus oocyte complexes obtained from the oocyte retrieval procedure were preincubated for 3 h (in a gas phase of 5% O_2_, 6% CO_2_, and 89% N_2_ at 37°C), followed by denuding the cumulus/oocyte complex cells. Oocytes in metaphase II subsequently underwent intracytoplasmic sperm injection (ICSI) and were cultured in a CCM‐IVF and iBIS (Astec) or EmbryoScope + time‐lapse incubator (Vitrolife). Fertilization was confirmed 16–18 h post ICSI, after which blastocysts developed over a maximum 6‐day incubation period in Global medium (LG; Life Global), covered with mineral oil (SAGE, Origio). Biopsies were performed on blastocysts that were elected to be discarded. Reasons for patient desire to discard were as follows: First, when a large number of embryos reach the blastocyst stage, we discarded some of them and did not freeze them (e.g., when a patient is not planning a second pregnancy and believes that a few good embryos are sufficient). Second, there are cases where a blastocyst is discarded due to payment issues. For example, some patients have a predetermined amount that they can afford to spend, and if they exceed that amount, they will decide against proceeding with the freezing of the blastocyst. There are also patients who wish to reduce the burden of annual frozen storage costs.

### 2.4 TE biopsy

The biopsies used the pulling and flicking methods. With the pulling method, blastocysts were held with the holding pipette and the biopsy pipette was used to pull TE cells away from the blastocyst while laser pulses were applied. With the flicking method, TE cells were drawn inside the biopsy pipette and subsequently excised with a quick movement of the biopsy pipette against the holding pipette.

Tissue cultures (Falcon 351007), equal to the number of embryos undergoing biopsy, were prepared in advance. After a holding pipette (Medicon International Co., Ltd.) and a 25‐μm biopsy pipette (Sunlight Medical) were set up, the blastocysts were transferred to biopsy tissue cultures. To prevent the adhesion of cells to the biopsy pipette, polyvinylpyrrolidone (Origio) was suctioned into and ejected from the pipette to coat its interior. TE biopsy was performed using the original method devised by our clinic.^20^ First, in embryos suspended with the holding pipette, laser irradiation (90 μs) was used from the 3 o'clock position to create a small opening in the zona pellucida (at a site containing more TE cells). Next, to remove the TE cells adhering to the interior of the zona pellucida, the culture medium was injected into the perivitelline space using a biopsy pipette. Afterward, the small opening in the zona pellucida was gradually expanded with laser irradiation (120 μs; LYKOS laser; Hamilton Thorne, Inc.) to make its diameter similar to the inner diameter of the biopsy pipette. The medium was injected into the opening to remove TE cells from the inner surface of the opening in the zona pellucida. Finally, extruded TE cells were collected. The zona pellucida was opened with laser irradiation at a heat range that would cause minimal damage to the cells (a pulse of 300 μs). Cell counts were performed by two or more embryologists, and only cells with visible nuclei were counted.

### 2.5 Tubing and NGS analysis of cell specimens obtained through TE biopsy

Tubing and NGS analysis of cell specimens obtained through TE biopsy were performed according to the method described by Takeuchi et al.^21^ Briefly, as a preliminary preparation, we entered the sample number in the dish (Falcon 351007) to wash the sample cells and a 0.2‐ml tube (Eppendorf polymerase chain reaction [PCR] tubes 0.2 ml) to store the sample. The Pasteur pipette tip was lightly washed with polyvinyl pyrrolidone (Origio) to prevent cell stickiness. After washing the sample three times with phosphate‐buffered solution, it was moved to the bottom of the 0.2‐ml tube, and the tube lid was closed. After centrifugation, the samples were stored at −20°C. The actual NGS procedures were as follows: First, the whole sample was subjected to whole‐genome amplification using a Veriseq PGS kit (Illumina) and a thermal cycler (Mastercycler Nexus, Eppendorf); the obtained sample was quantified and diluted to 0.2 ng/μl, and the diluted sample was amplified using a docosahexaenoic acid tag and PCR. After amplification, cleanup and normalization were performed, as well as load library formation, pooling and loading. The next day, the obtained data were subjected to chart analysis using BlueFuse Multi Software (Illumina).

### 2.6 Criteria for assessing aneuploidy in analysis results

Embryos were classified as euploid, mosaic, or aneuploid. The cut‐off point for mosaicism was defined as >20% of abnormal cells. Percentages <20 were classified as normal (euploid); >80, abnormal (aneuploid); and 20–80, mosaic.

### 2.7 Research designs

#### 2.7.1 Study I

We examined the effects of differences between operators on the occurrence of mosaicism. TE biopsies were performed using the pulling method. This study included 144 blastocysts from 26 patients who consented to have their embryos discarded, following the conclusion of PGT‐A clinical studies and culture from 2015 to 2019. In this study, we compared the results among three operators with more than 5 years of experience as embryologists, and who had undergone more than 1 year of training for biopsy techniques.

#### 2.7.2 Study II

We examined the effect of differences in TE biopsy techniques on the occurrence of mosaicism. In this study, which was conducted with eight embryos biopsied with the pulling method and demonstrated mosaicism in a previous diagnosis, we repeated the biopsy with the flicking method and investigated whether the occurrence of mosaicism changed. Notably, eight blastocysts were rebiopsied from Study I (wherein 144 blastocysts were initially investigated). After performing the biopsy using the pulling method, the cells were immediately frozen by CryoTip (Kitazato Corporation). The flicking method was used for re‐biopsy after 2–3 h of recovery culture post‐thawing.

#### 2.7.3 Study III

We examined the effect of differences in the number of cells collected on the occurrence of mosaicism. Biopsies were performed using the pulling method. We also determined the cut‐off number of collected cells to predict the occurrence of mosaicism. This study included 204 blastocysts from 18 patients who consented to have their embryos discarded (cultured from 2016 to 2019).

#### 2.7.4 Study IV

We collected cells according to the above cut‐off value (i.e., ≥5 cells) and compared the rates of euploidy, mosaicism, and aneuploidy, as well as the number of collected cells between biopsies performed with the pulling method and those performed with the flicking method. We attempted to collect at least five cells. In addition, cells were collected while checking with two or more embryologists to avoid arbitrary selection. This study included 201 blastocysts from 108 patients who consented to have their embryos discarded following the conclusion of PGT‐A clinical studies (cultured from 2020 to 2021).

### 2.8 Statistical analysis

We used BellCurve (version 3.20) for Excel (Social Survey Research Information Co., Ltd.) for all statistical analyses. Statistical analyses were performed using the chi‐square (*χ* ^2^) test with continuity correction, Mann–Whitney U test, and Kruskal–Wallis or one‐way analysis of variance. Receiver operating characteristic curves were used to determine the cut‐off value of the area in square microns, and statistical significance was set at *p *< 0.05.

## 3 RESULTS

### 3.1 Study I

There were no major differences in euploidy (14.8%–22.7%), mosaicism (14.8%–19.6%), or aneuploidy (60.6%–70.4%) among the three operators (Table 1).

**TABLE 1:** NGS analysis results of each operator

Operator	*n*	Age, years^a^	No. of euploidy	No. of mosaic	No. of aneuploidy
A	66	36.36 ± 2.91	15 (22.7%)	11 (16.7%)	40 (60.6%)
B	51	36.37 ± 2.97	8 (15.7%)	10 (19.6%)	33 (64.7%)
C	27	36.19 ± 2.73	4 (14.8%)	4 (14.8%)	19 (70.4%)

Abbreviation: NGS; next‐generation sequencing.

^a^ Data are presented as mean ± standard deviation.

### 3.2 Study II

**FIGURE 1:**
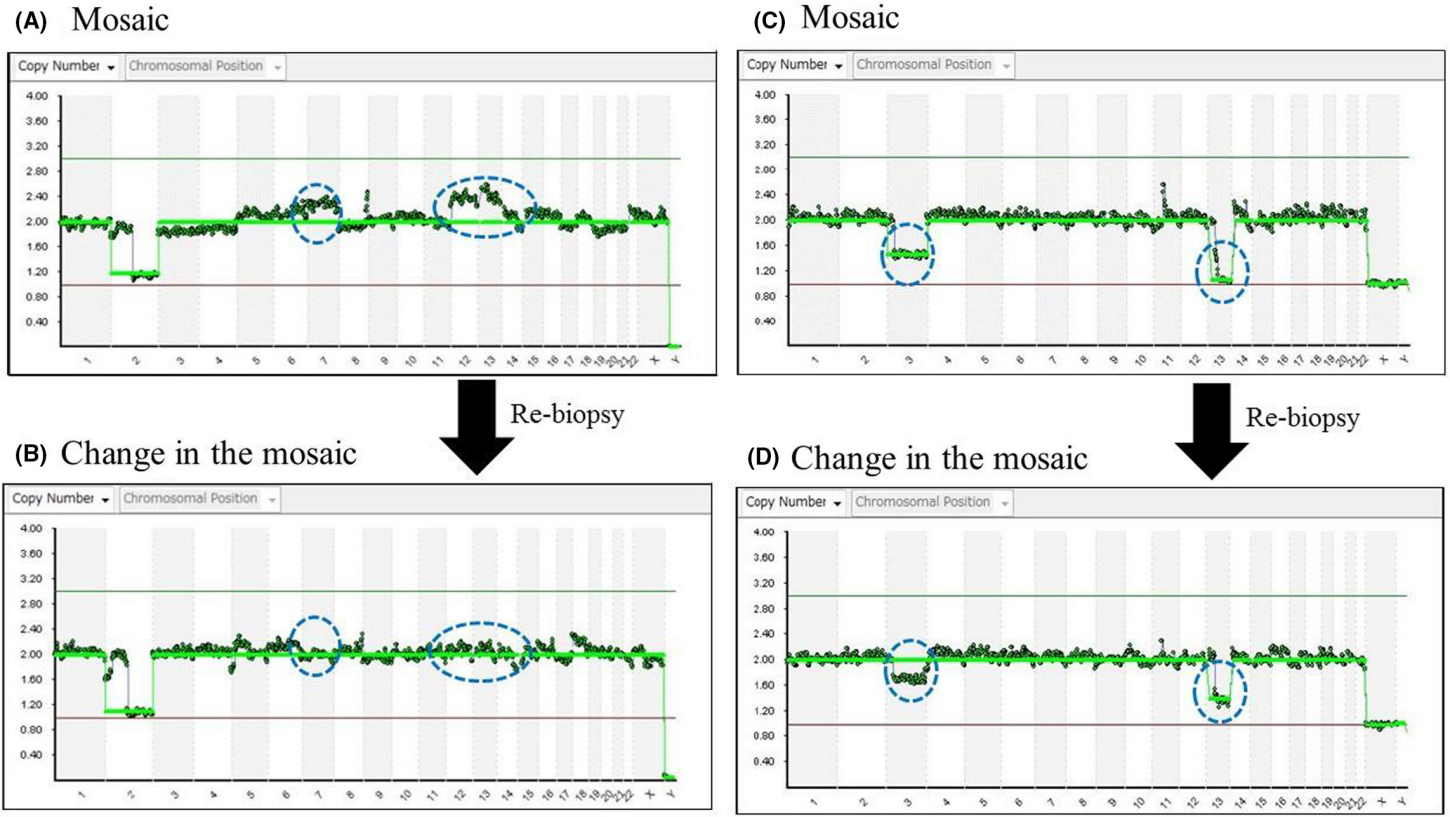
Actual next‐generation sequencing chart samples. (A and C) Mosaic. (B and D) Change in the mosaic after re‐biopsy

Mosaicism changed in all specimens (Figure 1). The number of collected cells with the flicking method (8.50 ± 2.27) was significantly higher than that with the pulling method (5.13 ± 0.99) (Table 2).

**TABLE 2:** Effect of differences in TE biopsy technique on the occurrence of mosaicism

No.	No. of collected cells		Results
	Pulling method	Flicking method	
1	5	6	Change
2	6	5	Change
3	4	8	Change
4	5	10	Change
5	7	8	Change
6	4	12	Change
7	5	10	Change
8	5	9	Change
Average^a^	5.13 ± 0.99*	8.50 ± 2.27**	

Note

*p < 0.01; **p < 0.01.

Abbreviation: TE; trophectoderm.

^a^ Data are presented as mean ± standard deviation.

### 3.3 Study III

**FIGURE 2:**
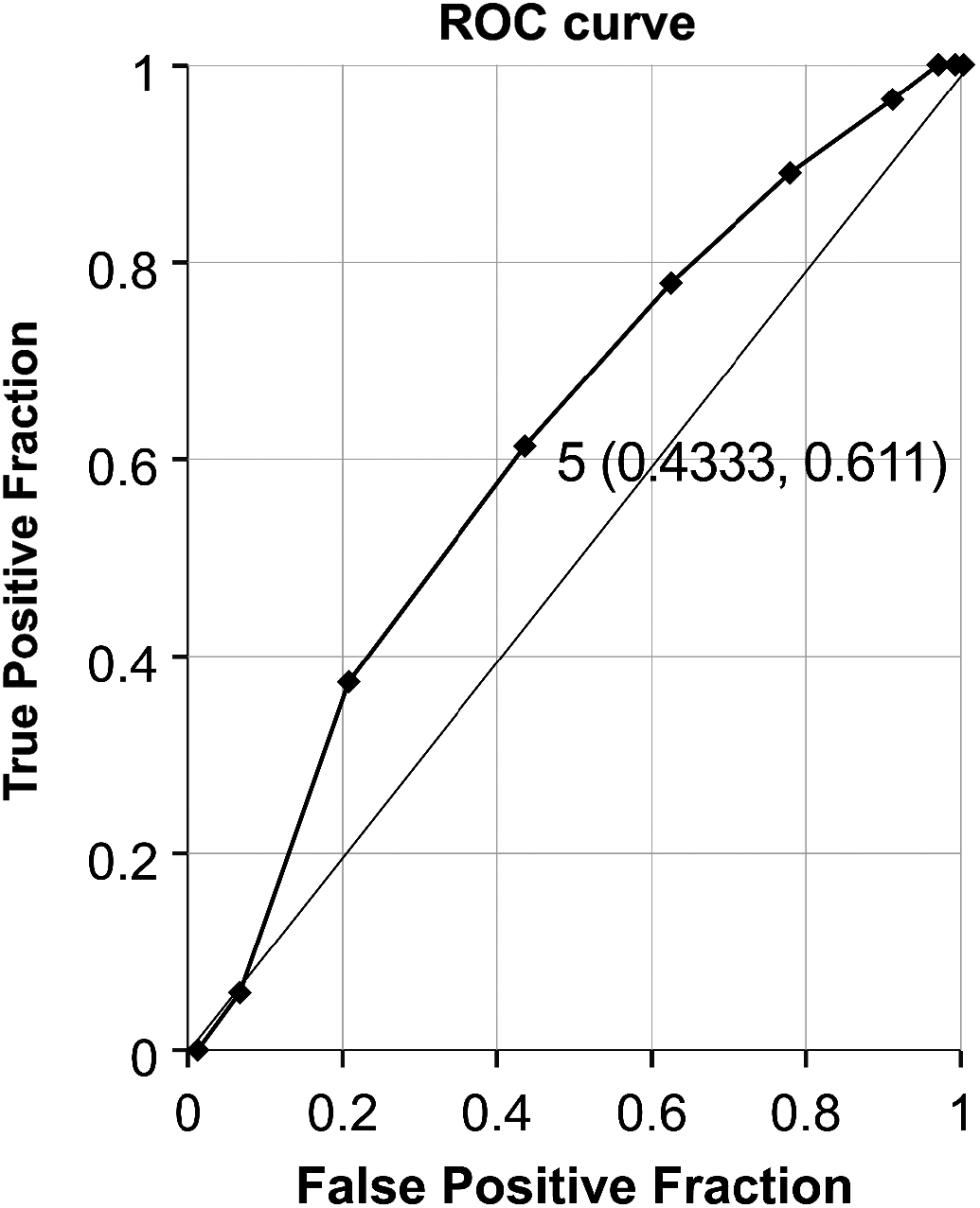
Cut‐off value of the number of collected cells to predict the occurrence of mosaicism. Area under the receiver operating characteristic curve (AUC) 0.6156, 95% confidence interval (CI) 0.5315–0.6997

The number of collected cells was significantly higher in the Mosaic (−) group (6.06 ± 1.95) than in the Mosaic (+) group (5.33 ± 1.53) (Table 3). The cut‐off value for the number of collected cells was five (Table 4; Figure 2).

**TABLE 3:** Effect of differences in number of collected cells on the occurrence of mosaicism

	*n*	No. of cells^a^
Mosaic (−)	150	6.06 ± 1.95*
Mosaic (+)	54	5.33 ± 1.53**

Note

*p < 0.05; **p < 0.05.

^a^ Data are presented as mean ± standard deviation.

**TABLE 4:** Calculation of cut‐off value of the number of cells collected at analysis for predicting the occurrence of mosaicism

*n*	Cut‐off value	Area under the ROC curve	*p* Value
204	5	0.6156	0.0071

Abbreviation: ROC, receiver operating characteristic.

### 3.4 Study IV

The number of collected cells did not differ significantly between the pulling (6.76 ± 1.41) and flicking (7.33 ± 2.02) methods. There were also no significant differences in the rates of euploidy (24.4%–26.9%), mosaicism (15.4%–15.6%), or aneuploidy (57.7%–60.0%) (Table 5).

**TABLE 5:** Effect of collection method on analysis results when collecting cells with the cut‐off value as a reference

Methods	No. of blastocysts	No. of cells	Analysis results		
			Euploidy (%)	Mosaic (%)	Aneuploidy (%)
Flicking	45	7.33 ± 2.02	11 (24.4)	7 (15.6)	27 (60.0)
Pulling	156	6.76 ± 1.41	42 (26.9)	24 (15.4)	90 (57.7)

## 4 DISCUSSION

Study I revealed that there were no differences between the operators and, in Study II, mosaicism changed in all specimens. The number of collected cells was significantly higher with the flicking method than with the pulling method. In Study III, the number of collected cells was significantly higher in the Mosaic (−) group than in the Mosaic (+) group, and the cut‐off value for the number of cells collected was five. In Study IV, the number of collected cells did not differ significantly between the pulling method and the flicking method. There were also no significant differences in the rates of euploidy, mosaicism, or aneuploidy.

The present study suggests that the analysis results in PGT‐A may be affected by the number of cells collected in the TE biopsy, that is, it may be affected by the location of cell collection. There have been similar reports regarding the location of cell collection.^22^ However, this finding is dependent on the absence of differences in the technique between operators and the stability of NGS analysis (i.e., not being dependent on multiple analysis centers). As Table 1 shows, the analysis results in the present study did not differ among operators. Approximately, 10%–20% of NGS‐based PGT‐A results are reported to be mosaic^15^, ^23^; the present study also yielded a mosaicism frequency of 10%–20%. In addition, all analyses in the present study were conducted at our clinic; thus, differences between centers (analysis at multiple centers) are an unlikely explanation. We believe that there were no problems with NGS analysis in the present study.

The results of Study II suggested that NGS analysis was affected by the frequency of changes in mosaicism occurrence based on the location of cell collection. At a glance, the flicking method appears to be associated with a lower frequency of mosaicism than that with the pulling method; however, the number of collected cells was significantly higher with the flicking method than with the pulling method. Based on this result, we compared the relationship between the occurrence of mosaicism and the number of collected cells, which may be affected by the location of cell collection; this comparison revealed that the number of collected cells was significantly lower among patients whose embryos demonstrated mosaicism than among patients whose embryos did not. These results showed that differences in the location of cell collection affected the NGS analysis results more than the biopsy technique, conceivably because the mosaicism rate changes with the location of cell collection. However, a study reported that, in NGS analysis results for re‐biopsy, concordance rates for mosaicism results were not very high.^24^ In the present study, NGS analyses after biopsy (first time) and re‐biopsy (second time) were stable; the only changes were those in mosaicism. There are three possible reasons for this finding. The first is the establishment of cryopreservation and thawing techniques. Our clinic has achieved favorable pregnancy rates with re‐cryopreserved and rethawed blastocyst transfer using CryoTip.^25^ Therefore, in the re‐cryopreservation and rethawing of blastocysts, biopsy can be performed without any decrease in the quality of blastocysts. The second reason is the TE biopsy technique. In the present study, NGS analysis did not yield any indeterminate results. One conceivable reason for this is that we could not collect a sufficient volume of cells for analysis during TE biopsy; another is that, while tubing, we could not transfer the biopsied cells into the sample tubes without failure. The last reason for the stability of our NGS analysis was that we performed it at our clinic. The ability to perform all procedures, from biopsy to analysis, at our clinic allowed us to control temperature and eliminate risks such as those associated with transport. Considering the above‐mentioned points, we can conclude that our NGS analysis results were affected by the location of cell collection.

The results in Table 1 show that there were no major differences in NGS results between operators. Factors such as time taken for the TE biopsy and laser irradiation did not affect NGS results. In addition, since only fresh blastocysts from Day 5, which the patients requested to be discarded, were included in the study, there was no effect from incubation time. Based on these assumptions, we conducted study III. In TE biopsy, the necessary volume of TE cells is suctioned into a biopsy pipette, and the cells are then generally collected using one of two methods: the pulling‐stretching method, in which the cells are dissected with laser pulses; and the flicking method, in which cells are dissected with a flicking movement of the biopsy pipette against the holding pipette. In Study III of the present study, the cut‐off value of the number of collected cells to predict the occurrence of mosaicism was five. This suggests that if more than five cells are collected during TE‐biopsy, stable NGS analysis results can be obtained. Based on this result, we examined differences in NGS analysis results between the methods (pulling or flicking) in Study IV. As Table 5 shows, in the NGS analysis conducted after a target number of collected cells was set, results of the flicking and pulling methods were similar. This demonstrates that differences in collection methods did not affect NGS analysis results if the number of collected cells was equal and suggests that differences in the number of cells collected in TE biopsies affect NGS analysis results.

In addition to improved therapeutic outcomes and shortened treatment times, another merit of PGT is the reduced transfer of multiple embryos and the increased transfer of single embryos. This can be expected to reduce the risk of complications associated with multiple pregnancies.^15^ However, PGT is not recommended for all infertile women.^26^, ^27^, ^28^ In addition, several studies have not demonstrated that PGT‐A improves live birth rates.^29^, ^30^, ^31^ In PGT, treatment is sometimes discontinued without blastocyst development, and euploid embryos sometimes cannot be obtained even if a developed embryo is biopsied.^32^ Rather than performing PGT for all patients, it should be performed only for patients who need it.

The present study demonstrated that if TE biopsy techniques and NGS analysis are stable, the location of cell collection has a greater effect on NGS analysis than the embryo biopsy technique. Embryos should be cultured until more trophectoderm cells (at least five) can be obtained. In the future, we will attempt to optimize embryo culture, biopsy methods, and analysis procedures to obtain more stable NGS analysis results.

## CONFLICT OF INTEREST

The authors declare that they have no conflict of interest.

## ETHICAL APPROVAL

The study was performed with the approval of the institutional review board of the Takeuchi Ladies Clinic. Opt‐out information was posted on a hospital bulletin board.

## HUMAN RIGHTS STATEMENTS AND INFORMED CONSENT

All procedures were performed in accordance with the ethical standard of the responsible committees on human experimentation (institutional and national) and with the Helsinki Declaration of 1964 and its later amendments. Informed consent was obtained from all patients in the study.
